# Understanding the fundamentals of oscillometry from a strip of lung tissue

**DOI:** 10.3389/fphys.2022.978332

**Published:** 2022-09-20

**Authors:** Ynuk Bossé

**Affiliations:** ^1^ Institut Universitaire de Cardiologie et de Pneumologie de Québec—Université Laval, Québec, QC, Canada

**Keywords:** impedance, lung tissue, oscillometry (forced oscillation technique), respiratory mechanics, pulmonary physiology

## Abstract

Metrics used in spirometry caught on in respiratory medicine not only because they provide information of clinical importance but also because of a keen understanding of what is being measured. The forced expiratory volume in 1 s (FEV_1_), for example, is the maximal volume of air that can be expelled during the first second of a forced expiratory maneuver starting from a lung inflated to total lung capacity (TLC). Although it represents a very gross measurement of lung function, it is now used to guide the diagnosis and management of many lung disorders. Metrics used in oscillometry are not as concrete. Resistance, for example, has several connotations and its proper meaning in the context of a lung probed by an external device is not always intuitive. I think that the popularization of oscillometry and its firm implementation in respiratory guidelines starts with a keen understanding of what exactly is being measured. This review is an attempt to clearly explain the basic metrics of oscillometry. In my opinion, the fundamentals of oscillometry can be understood using a simple example of an excised strip of lung tissue subjected to a sinusoidal strain. The key notion is to divide the sinusoidal reacting force from the tissue strip into two sinusoids, one in phase with the strain and one preceding the strain by exactly a quarter of a cycle. Similar notions can then be applied to a whole lung subjected to a sinusoidal flow imposed at the mouth by an external device to understand basic metrics of oscillometry, including resistance, elastance, impedance, inertance, reactance and resonant frequency.

## Introduction

Oscillometry is practical because it is effortless and requires very little cooperation, making it easy to perform for both the subject and the operator. This also means that, in contrast to spirometry, it can be performed in a reliable fashion on infants and children (Gray et al., 2019; Lavizzari et al., 2021; Ducharme et al., 2022), as well as on patients who are very sick (*i.e.*, weak) or afflicted by disabilities (Veldhoen et al., 2022; Vielkind et al., 2022) or other conditions where forced expiratory maneuvers are contraindicated (Jordon et al., 2022). Oscillometry is also useful for many reasons. First, it has a fine time-resolution (number of data points per unit of time) meaning that the kinetics of events occurring over smaller time-scales can be measured. This feature is extensively exploited in respiratory research to study the kinetics of bronchoconstriction, the bronchodilator effect of a deep inspiration, and the rate of airway renarrowing post-deep inspiration (Jensen et al., 2001; Salome et al., 2003; Jackson et al., 2004; Gobbi et al., 2013; Chapman et al., 2014; Gazzola et al., 2020), as well as to develop new diagnostic tests to assess, for example, expiratory flow limitation (Dellaca et al., 2004; Dellaca et al., 2007) or airway distensibility (Brown et al., 2004; Brown et al., 2007; Kelly et al., 2010; Mailhot-Larouche et al., 2017). Secondly, oscillometry provides topographical information, in particular to the respective contribution of large versus small airways in lung dysfunction (Hantos et al., 1992; Suki et al., 1997; Takeda et al., 2010; Shi et al., 2012; Berger et al., 2016; Chiu et al., 2020; Bhatawadekar et al., 2021; Pisi et al., 2021). Finally, many even propose that oscillometry provides complementary insights to spirometry to predict and diagnose patients with various respiratory disorders (Goldman et al., 2002; Shi et al., 2012; Kelly et al., 2013; Shi et al., 2013; Berger et al., 2016; Jabbal et al., 2016; Karayama et al., 2018; Bellisario et al., 2019; Chiu et al., 2020; Salvi et al., 2020; Chaiwong et al., 2021; Chiabai et al., 2021; Pisi et al., 2021; Accorsi et al., 2022; Takahashi et al., 2022). Despite all these assets, the current rate of implementation of oscillometry in respiratory medicine is rather slow. I also have the feeling that many clinicians, generalists and respirologists alike, are sometimes reluctant to adopt it as a routine test in their clinic. I think one major hurdle is the lack of understanding of what exactly is being measured.

Indeed, oscillometry is often perceived as a daunting science for newcomers. Several metrics are used, including resistance, conductance, elastance, compliance, impedance, admittance, reactance, susceptance, inertance and resonance. To add to this complexity, experts in respiratory mechanics refer to these metrics as being either real or imaginary. Resistance, for example, is typically real, while reactance is imaginary. Some metrics are even both, real and imaginary, such as impedance. This review borrows the shortest path possible to explain the basic metrics of oscillometry and why they can be considered either real or imaginary.

In the first section, I discuss about a strip of lung tissue subjected to a low-amplitude sinusoidal strain in an *in vitro* setting. This very simple example illustrates how the practicalities of the sine wave can be exploited to extract elastance and resistance from the resulting reacting force. In the second section, I then build on these notions to explain how a low-amplitude sinusoidal flow signal delivered through the mouth by an external device, together with the resulting change in mouth pressure, can be used to deduce the same metrics (elastance and resistance). In this latter *in vivo* setting, it will also become apparent that other metrics need to be understood, including impedance, inertance, reactance and resonance.

## Resistance and elastance in an isolated strip of lung tissue

Imagine a pendulum with its bob swinging left and right. On one hand, when the bob is at the extreme positions in relation to the horizontal plan, either at the farthest left or right, its velocity is zero. It is actually turning around at those very moments. On the other hand, when the bob is at the middle position in relation to the horizontal plan, its velocity is maximal, irrespective of whether it is moving towards the right or left. Therefore, when the position of the bob and its velocity in relation to the horizontal plan are plotted as functions of time, the sine wave defining its position at any given moment and the sine wave defining its velocity at the same corresponding moment are phase-shifted by exactly 90° (*i.e.*, a quarter of a cycle).

**FIGURE 1:**
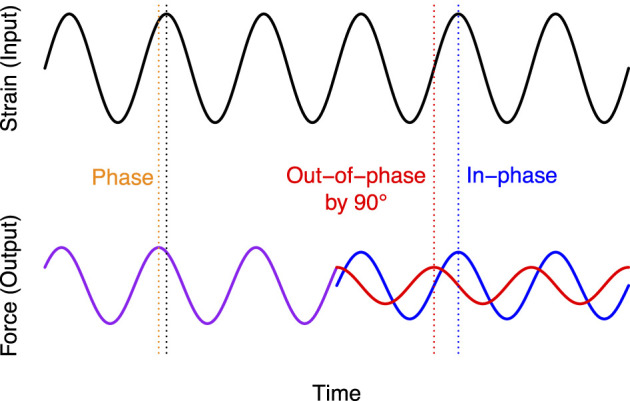
Exploited practicalities of the sine wave in oscillometry. The sine wave on top (black) is the input signal; *i.e.*, the one controlled by the experimenter. For example, the strain (in mm) on an excised strip of lung tissue. The purple line on the bottom left is the output signal; *i.e.*, the one measured by the experimenter. In this example, the output signal would be the force (in mN). Note that the sine wave shape and the frequency of the oscillation were preserved from the input to the output (*i.e.*, the black and purple lines are sine waves at the same frequency). However, the output sine wave has changed in amplitude and phase (orange) in relation to the input. These are convenient practicalities of the sine wave that pertain as long as the material being investigated behaves linearly. It means that the response of a linear system to a sinusoidal oscillation is simply characterized by measuring how the sine wave amplitude and phase are changing from the input to the output. Another convenient practicality of the sine wave is that by adding two sine waves of identical frequency together, the result is always another sine wave at the same frequency. Based on the same reasoning, the output in purple may originate from two sine waves, as shown in blue and red on the bottom right. Note that these two sine waves are again at the same frequency. Also note that while the blue was set to be in-phase with the input signal (*i.e.*, strain), the red was set to be out-of-phase from the blue, as well as the input signal, by exactly 90°. The red sine wave is thus a cosine wave. Importantly, when the strain rate (*i.e.*, the first derivative of strain) is calculated from the input signal, the amplitude of this rate over time would follow a sine wave in-phase with the red line. The strain and the strain rate are thus out-of-phase by exactly 90° when the strain follows the form of a sine wave. As discussed in the text, the elastic and resistive forces emanating from the tissue during sinusoidal straining are also in-phase with the strain and the strain rate, respectively. Therefore, dividing a sinusoidal output signal (purple) into a component in-phase with the input (blue) and another component 90° out-of-phase with the input (red) is an easy trick that simultaneously separates the respectively contribution of elastance and resistance. Essentially, it means that the forces attributed to elastance and resistance over time are described by the blue and red lines, respectively.

The same applies between the strain and the strain rate for a lung tissue strip being alternately stretched and retracted sinusoidally (Figure 1). When the tissue is strained from a position of −1 to 1, the strain rate is maximal when the position is zero, while the maximal amplitude (position 1) corresponds to the point where strain rate is back to zero. The sine wave defining the strain rate thus precedes the sine wave defining the strain by exactly 90°.

Now, during sinusoidal straining, the reacting force (*i.e.*, the output signal) from the tissue fluctuates. As long as the tissue behaves linearly (this essentially means that the relationship between strain and elastic force, as well as between strain rate and resistive force, would be described by a straight oblique line), this total reacting force should also take the form of a sine wave. This reacting force can then be divided into two forces. One in-phase with the strain, which is called the elastic force, and another one in-phase with the strain rate, which is called the resistive force. In fact, as illustrated in Figure 1, the total force at the output is predicted by a relatively simple equation:

$$F\left(t\right)=E\epsilon +R\epsilon^{\prime }$$ (1)

Where F is force, E is elastance, R is resistance, ε (epsilon) is strain and ε' (epsilon prime) is strain rate. The letter t between parentheses stands for time, and indicates that this function describes the change in force over time. Since force is measured and ε and ε′ are the ones prescribed by the experimenter, the only missing values in this equation are E and R. Their best fits to the force data can be deduced by multiple-linear regression, assuming again that the lung tissue behaves linearly and, thus, E and R are constants. I want to emphasize that, in reality, E and R are not constants. It is understood that their values are affected by strain (*i.e.*, initial level of stretch) (Ito et al., 2006). However, at any starting strain, and over a small range of sinusoidal strain, it can be assumed that E and R are virtually constants and that the lung tissue then behaves linearly. This is why the imposed sinusoidal strains that are typically used to probe lung mechanics are of small amplitudes

Eq. 1 can also be defined equivalently in the frequency domain. The equation is then:

F(ω)=(E+iωR)ϵ (2)

Where ω (omega) is angular frequency. Its appearance between parentheses on the left-hand side of the equation indicates that this function describes the change in force over frequency. Angular frequency is more precisely equals to 2π the regular frequency (often abbreviated 2πf). 2π being a circle, 2πf is kind of a speed of rotation (or an angular displacement per unit of time). Multiplying ω with ε provides the strain rate, which, as explained in Figure 1, is in-phase with the contribution of resistance to the total force output. It can thus be seen from Eq. 2 that the process of differentiation in the frequency domain is very easy. In the time domain, the strain rate is deduced by calculating the first derivative of strain (the input signal) and, in the frequency domain, this is simply done by multiplying with iω. It will be seen later that the process of integration in the frequency domain is just as easy, being done by multiplying with -i/ω.

**FIGURE 2:**
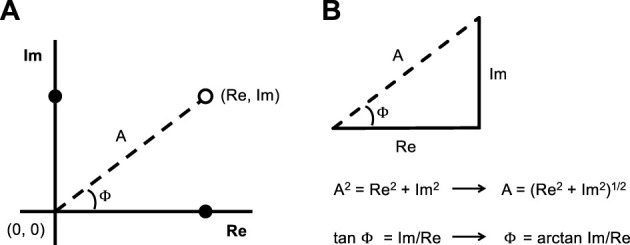
A complex plane is depicted in **(A)**, with its real (Re) and imaginary (Im) axes. The solid circle on each of these axes represent the magnitude of the real and imaginary parts of the output signal. When a straight line is traced from their Cartesian coordinates (Re, Im) (open circle) to the origin (0,0), it forms a vector (dashed line) with a length A and an angle ϕ (phi) that respectively define the required amplitude-scaling factor and the phase shift to transform the input sine wave (black sine wave in Figure 1) into the output sine wave (purple sine wave in Figure 1). This vector, combined with the magnitude of the real and imaginary parts, create a triangle that is depicted in **(B)**. It is then shown how A and ϕ can be calculated using the Pythagorean theorem and the tangent function of a right triangle using the values of the real and imaginary parts.

The factor i in the resistive term (iωRε) of Eq. 2 is the unit of imaginary numbers, or the square root of -1. An i in front of a number means that this number is imaginary. The right-hand side of Eq. 2 is thus a complex number, composed of a real part (Eε) and an imaginary part (iωRε). Complex numbers are used in the frequency domain because they represent an alternative equivalence of the cosine and sine components of the output signal in the time domain (Figure 1). The principle is not very complicated, provided a working knowledge with complex numbers and trigonometry. Briefly, on a complex plane, numbers are presented in two independent dimensions (Figure 2A). While the *x*-axis represents the dimension containing real numbers, the *y*-axis represents the dimension containing imaginary numbers. Multiplying by i effectively shifts the whole term (iωRε in Eq. 2) by 90° on a complex plane, appropriately indicating that the contribution of resistance to the output signal is out-of-phase by 90° in relation to the input (Figure 1). The magnitude of the vertical displacement on this complex plane thus represents the imaginary part of the output signal, and is used to quantify the contribution of the resistive term in Eq. 2 (iωRε). In the time domain, this vertical displacement would correspond to a factor that adjusts the amplitude of the cosine wave. In turn, the magnitude of the horizontal displacement on this complex plane represents the real part of the output signal, which is used to quantify the contribution of the elastic term in Eq. 2 (Eε) and would correspond to a factor that adjusts the amplitude of the sine wave in the time domain. Note that tracing a straight line from the origin to the Cartesian coordinates given by the real and imaginary numbers (Re, Im) forms a vector with a length and angle that can be calculated according to the Pythagorean theorem and basic trigonometry (Figure 2B). Recall from Figure 1 that the response of a linear system to a sinusoidal oscillation is fully characterized by measuring the factor by which the output sine wave amplitude is scaled and the amount by which its phase is shifted in relation to the input sine wave. The length and angle of the calculated vector above actually provide these entities; the length of the vector defines the extent by which the sine wave has been scaled in amplitude (the output amplitude divided by the input amplitude) and the angle defines the amount by which it has been shifted in phase (the output phase minus the input phase). This is why the combination of a real and imaginary numbers can be used, just as effectively as an amplitude (length of a vector) and a phase (the angle of a vector), to quantify the response to a sinusoidal oscillation.

One common source of confusion when first introduced to these concepts is the redundant terminology. Be aware that the cosine wave (Rε′ in Eq. 1), representing the out-of-phase component of the output signal in the time domain (Figure 1), can be alternatively called the imaginary part because it is calculated using the imaginary unit in the frequency domain (iωRε). Concordantly, the sine wave (Eε in Eq. 1), can be called either the in-phase component or the real part of the output signal because it is in-phase with the input in the time domain and also depicted in the real dimension on the complex plane in the frequency domain.

## Oscillometry

The guiding principles of oscillometry when it is used to probe the mechanics of the respiratory system in humans is not very different from the ones used to calculate elastance and resistance from a strip of lung tissue strained sinusoidally. Of course, resistance and elastance of the whole respiratory system stem from additional origins than the tissue. Both are also affected by the mechanical properties of the chest wall. Another source of resistance stems from the airflow within airways. Yet, the way resistance and elastance is calculated is the same. In fact, all equations outlined above are applicable for a respiratory system being strained sinusoidally. Eq. 1 is then substituted by:

$$P\left(t\right)=EV+RV^{\prime }$$ (3)

where P is pressure (*i.e.*, a force applied in three dimensions), E and R are still resistance and elastance, V is volume (*i.e.*, strain in three dimensions) and V′ is flow (*i.e.*, the rate of change in volume over time, or the strain rate in three dimensions)

For sinusoidal strain, Eq. 3 can also be defined equivalently in the frequency domain. The equation is then:

P(ω)=(E+iωR)V (4)

As per the transformation of strain into strain rate in Eq. 2, note here that R is multiply by iω, since it involves a differentiation in the frequency domain. This is because resistance is related to flow, and flow in the time domain can be deduced by calculating the first derivative of volume (the input signal).

Importantly, it is customary to use flow, instead of volume, as the input signal in oscillometry. When flow is used as the input, Eq. 4 becomes:

$$P\left(\omega \right)=\left(R-iE/\omega \right)V^{\prime }$$ (5)

Note here that E is multiply by -i/ω, since it involves an integration in the frequency domain. This is because elastance is related to volume, and volume in the time domain can be deduced by integrating flow (the input signal).

The last equation more often takes this form:

$$P\left(\omega \right)/V^{\prime }=R-iE/\omega =Z\left(\omega \right)$$ (6)

Where the two measured variables (pressure and flow) are on the left-hand side of the equation and Z is impedance.

One common perplexing observation is that resistance is sometimes imaginary (Eqs 2, 4) and sometimes real (Eq. 5). In the example shown in Figure 1, resistance is the imaginary part, while elastance is the real part. Contrastingly, in oscillometry, resistance is conventionally real. However, this is only determined by the input signal. In the example in Figure 1, where a strip of lung tissue is strained sinusoidally, the strain amplitude is the input. Since the contribution of elastance to the total force is in-phase with the input, it is then considered the real part. The contribution of resistance, on the other hand, is 90° out-of-phase from the input and is then considered the imaginary part. If someone had used the strain rate, instead of the strain amplitude, for the input signal, then the resistance would have been real and the elastance imaginary. The same applies to oscillometry, where flow is customarily used as the input signal. Since the contribution of resistance to the total pressure is in-phase with the input flow, it is thus considered the real part. The contribution of elastance, on the other hand, is 90° out-of-phase from the input and is thus considered the imaginary part. If someone had used the volume, instead of the flow, for the input signal, then the elastance would have been real and the resistance imaginary. Hopefully, it can be deduced from this explanation that the real and imaginary parts are both very real. The term “imaginary” carries this inopportune connotation of being less applicable to our real world, but this is irrelevant. The part of impedance that is considered imaginary can be allocated to either elastance or resistance indistinctly depending on whether the imaginary unit is used to calculate one or the other.

It is also worthy to mention at this point that another reacting force, called inertance, is sometimes important to consider. Its magnitude is influenced by the change in strain rate; viz. strain acceleration. When the strain follows the form of a sine wave, strain acceleration is out-of-phase by 90° in relation to the strain rate and by 180° in relation to the strain. Imagine the pendulum again. When the bob’s position is moving upward and downward, it is decelerating and accelerating, respectively, due to gravity. When the bob reaches its farthest position on the left, the gravitational force pulling it back to the right is maximal. Alternatively, when the bob reaches its farthest position on the right, the gravitational force pulling it back to the left is maximal. At the very mid position in relation to the horizontal plan (*i.e.*, the zero position because neither left nor right), the gravitational force pulling the bob left or right is also zero. Therefore, the position and the acceleration of the bob in relation to the horizontal plan are exactly 180° out-of-phase to each other. To put it in a context of a lung during an input flow signal, the change in pressure due to resistance (*i.e.*, the in-phase component) lags the change in pressure due to inertance by 90°, while it leads the change in pressure due to elastance by 90°.

The change in pressure (or force) due to inertance can often be neglected, because its overall contribution is sometimes very small, especially at low frequencies. This is the case for a lung tissue strip being studied *in vitro*. It is also the case for the lung of small animals, such as mice (Gomes et al., 2000). However, for the lung of large mammals, including humans, inertance is contributing significantly to the total change in pressure. The inertance of the lung is chiefly attributed to the change in pressure that is required to accelerate the enclosed air in conducting airways. Regardless, the notion I want to emphasize here is that, because elastance and inertance are out-of-phase by 180°, they cannot be dissociated alike elastance and resistance in Figure 1. Elastance and inertance rather tend to cancel each other out. In fact, when their magnitude is identical, they completely cancel each other out. The frequency at which this occurs is called the resonant frequency. At the resonant frequency, the impedance is merely the resistance. At any other frequencies, the sine wave that is 90° out-of-phase from the input is a combination of elastance and inertance and is then called reactance. This is why people working in the field of oscillometry refer most commonly to reactance than elastance.

When inertance needs to be taken into account, Eq. 6 becomes:

Z(ω)=R+i(ωI−E/ω) (7)

Where I stands for inertance. Note that I is multiply by iω, since it involves a differentiation in the frequency domain. This is because inertance is related to the change in flow rate, and the change in flow rate in the time domain can be deduced by differentiating flow (the input signal).

This last equation is more commonly abbreviated as follows:

Z(ω)=R+iX (8)

where X is reactance.

## Conclusion

This review first explained how the practicalities of the sine wave can be exploited to calculate elastance and resistance in a strip of lung tissue subjected to a low-amplitude sinusoidal strain. This simple example, together with the involved principles, were then used to understand the fundamentals of oscillometry. It was by looking at the equation defining the pressure over flow in the frequency domain (Eq. 6) that the concept of impedance was introduced. Inertance was then taking into account and it was seen that it sums up with elastance to give reactance. Note that conductance, compliance, admittance and susceptance were not discussed as they are merely the inverse of resistance, elastance, impedance and reactance, respectively. Together, these represent the basic metrics used to describe lung mechanics by oscillometry.

The comparison with the lung tissue strip was also useful to understand why some metrics are considered imaginary. It was realized that resistance is typically imaginary when a lung tissue strip is studied in an *in vitro* setting, while it is rather typically real when the whole lung is studied in an *in vivo* setting. Thereby, it was understood that the use of a real and imaginary parts in the frequency domain merely provides an alternative to represent the sine and cosine components of the output signal in the time domain. Not only resistance, but other metrics in oscillometry can either be real or imaginary. The term imaginary is simply allocated to the part of the total output signal that contains the imaginary number, which, in turn, is determined by the part that is 90° out-of-phase with the input. In oscillometry, resistance is typically real because it is in-phase with flow, while elastance, inertance and reactance are imaginary because they are out-of-phase with flow. Finally, impedance is both, because it contains both the real and imaginary parts.

Notwithstanding the simplicity with which oscillometry was explained herein, it is important to keep in mind that it can be vastly more complicated. The input signal does not have to be applied at the mouth and it does not have to be a single-frequency sine wave. In fact, it is more often a broadband signal (*i.e.*, a signal made up of several sine waves at different frequencies, amplitudes and phases). It can even be a random signal that looks nothing like sine waves. It is actually in those circumstances that it becomes useful to look at impedance in the frequency domain. Comprehensive reviews on this topic were previously published, which also expatiate on the various mathematical tools that are required to process these different signals (LaPrad and Lutchen, 2008; Bates et al., 2011; Kaczka and Dellaca, 2011; Lappas et al., 2013). Yet, the calculated metrics are the same and, irrespective on how they are calculated, they carry essentially the same bearings for lung mechanics. The goal herein was to use the simplest and shortest path to clearly explain these basic metrics.

It should also be kept in mind that commercial devices are now readily available. Therefore, although the mathematical gymnastic involved may seem burdensome, these kinds of measurements are now readily accessible to anyone with or without a strong background in respiratory mechanics. Standardized procedures to perform oscillometry in humans were also recently published (King et al., 2020). As alluded to in the Introduction, these guidelines, combined with commercial devices, has now made oscillometry easier to apply in clinic. Research has also now confirmed that oscillometry provides highly sought-after physiological metrics that can be readily measured, that are altered in lung disorders, and that are modifiable in response to interventions. Parenthetically, its wide implementation in respiratory medicine would also facilitate translational research, as oscillometry is the technique of choice to study lung alterations in animal models of human diseases (Bates, 2017; Lundblad and Robichaud, 2021). Despite instrumentation availability, oscillometry is still underexploited and catches on at a rather slow pace in respiratory medicine. I think this is, in part, because its various metrics are misunderstood. I hope this review has helped potential users to understand what exactly is being measured and will encourage newcomers in this vibrant field.
